# The influence of differential leadership on employees’ deviant innovation behavior: An outsider subordinate perspective

**DOI:** 10.3389/fpsyg.2022.996331

**Published:** 2022-11-17

**Authors:** Mengyun Wu, Yuqing Zhu, Qi He, Luan Zhang, Jie Lu

**Affiliations:** ^1^School of Finance and Economics, Jiangsu University, Zhenjiang, China; ^2^School of Management, Jiangsu University, Zhenjiang, China

**Keywords:** differential leadership, deviant innovation behavior, relative deprivation, internal control, outsider subordinate perspective

## Abstract

Given the complex business environment worldwide and rapid development of information technologies, employees’ deviant innovation behavior has attracted increasing attention. However, few scholars have explored the internal mechanism of the influence of differential leadership on the deviant innovation behavior of outsider subordinates from a positive perspective. Based on relative deprivation theory and attribution theory, we construct a theoretical model to study the influence of differential leadership in family businesses on the deviant innovation behavior of outsider subordinates, and hypothesis testing is conducted based on 243 questionnaire responses. The result shows that: differential leadership has a positive impact on the deviant innovation behavior of outsider subordinates; relative deprivation plays a mediating role; and internal control personality enhances the moderation effect between relative deprivation and outsider subordinates’ deviant innovation behavior. This study provides a reference point for the promotion of the innovation performance both of employees and organizations.

## Introduction

Innovation behavior has always been widely regarded as an inexhaustible driving force in the development of organizations. However, in recent years, enterprises have gradually found that the innovation behavior of employees has become deviant, and that there is a potentially close and interesting relationship between innovation activities and workplace deviance (Deng, 2019). Although there are differences in defining deviant innovation behavior, scholars concur that the original intention of such behavior is not deviance but innovation (Criscuolo et al., 2014). Deviant innovation behavior aims to improve the benefits and performance of organizations, but it is not approved of by the leadership and has a negative effect on legitimacy, so it is sometimes conducted in a private and secret manner (Masoudnia and Szwejczewski, 2012). However, in the development of enterprises worldwide, many innovations, such as the Sogou browser, 3M scotch tape, and HP’s new monitor, were have been created by the deviant behavior of employees, entailing disruptive effects on the organizations. Particularly in the era of innovation-oriented development, organizations are paying much more attention to employees’ innovation achievements than the manner of innovation, which intensifies the contradiction between the search for innovation and the constraints of organizational systems, thus resulting in more deviant innovation behaviors (Wu et al., 2020). Deviant innovation behavior appears to violate organizational norms, but it can help enterprises to achieve the maximum efficiency of resources and break innovation bottlenecks if they are able to enhance its positive effects and reduce the negative effects through scientific guidance. Deviant innovation behavior thus represents a new and effective means of organizational innovation (Deng, 2019). Christensen et al. (2007) first proposed the concept of disruptive innovation and used it to describe innovations that disrupt the competitiveness of incumbent firms in existing mainstream markets. Deviant innovation behavior and disruptive innovation behavior are concepts that originated in the category of social psychology. Both of these two behaviors are important ways to promote employee innovation in the new era by breaking organizational routines. The purpose of both is to help enterprises achieve innovation and create greater value. Disruptive innovation pays more attention to the degree of realization, and effective leadership promotion is the key to the success of disruptive innovation; deviant innovation pays more attention to the way of realization, employees increase their motivation to participate in the realization of organizational goals through motivation, and take the initiative to develop their potential to improve participation in the realization of organizational goals to improve the overall innovation performance of the organization.

Previous research has focused on the consequences of deviant innovations, but research on its antecedents is lacking (Wang et al., 2018). Especially in China, it is worth studying how different leadership styles influence employees’ deviant innovation behavior (Guo and Li, 2015). Differential leadership is more susceptible to the psychological influence of “insiders” and “outsiders” and treats employees differently (Ruan, 2018). This kind of leadership style, with strong partiality, will affect the perceived fairness and innovation behavior of subordinates. Accordingly, questions such as why it has survived so long in Chinese enterprises and what impacts it will have on employees’ deviant innovation have attracted intense academic attention. Against the background of innovation-driven development strategy, the need to understand China’s own leadership values and to explore their impacts on employees’ deviant innovation, and subsequently on organizations’ innovation performance, has become necessary in the context of management localization.

Most current research has focused on the positive effects of differential leadership on insider subordinates (Zhao, 2019). In contrast, research on outsider subordinates has mostly focused on the negative effects (Liu et al., 2020), while ignoring the positive effects. For outsider subordinates, when employees are moderately in a state of dissatisfaction and anxiety, striving hard will become the motivation to stimulate their innovation behavior, thus prompting outsider subordinates to make achievements through deviant innovations (Weng, 2014). Research on employees’ perception of work situations suggests that, when the leaders treat subordinates differently inside organizations, employees who are treated unfairly often experience feelings of relative deprivation (Wan et al., 2016). A feeling of relative deprivation incurred by unfairness makes employees, out of the need for pressure release, more inclined to seek self-actualization through improving innovation ability and to try to move to groups of higher social status (Smith et al., 2012). Therefore, as a subjective perception of employees, relative deprivation is more likely to trigger their deviant innovation behaviors (Kinicki and Vecchio, 1994). Accordingly, this study introduces relative deprivation as a mediator variable and explores the internal mechanism of the influence of differential leadership on employees’ deviant innovation behavior. In addition, based on attribution theory, employees with internal control personality are more confident about the impact of self-abilities on the work environment, and they will give full play to initiative at work, thus reducing their feelings of relative deprivation and maximizing the management efficiency of organizations (Ke and Sun, 2018).

This study contributes to the current leadership, human resource management, and enterprise change management literature by formulating original hypotheses to reveal the impact of differential leadership styles on employees’ deviant innovation behaviors. Using social exchange theory, relative deprivation theory, and attribution theory, the scale design was carried out, and exploratory factor analysis, confirmatory factor analysis, descriptive statistics and correlation analysis, Bootstrap test, “moderated mediation test and other methods were used to obtain.” The theoretical model of the positive impact of differential leadership on employees’ deviant and innovative behaviors from the perspective of outsiders The internal relationship between leadership style and employees’ deviant innovative behaviors, discussing employees’ deviant innovative behaviors and their governance countermeasures, and clarifying the importance of flexible adjustment of leadership styles to effectively manage employees’ deviant innovative behaviors.

The remainder of this paper is structured as follows: the next section describes the theoretical support of this research and the development of the hypotheses tested in this study. Next in the research design, presents data resource, and statistical model and software that was used for testing the hypotheses. Subsequently, the empirical findings of the proposed hypothesized model are presented. The paper concludes with a summary of the important findings, limitations of the study, and directions of future research to develop this burgeoning area of organizational change management.

## Theoretical background and hypotheses

### Differential leadership and outsider subordinates’ deviant innovation

The relationship between a leader and subordinates is dynamic. Leaders will judge insider and outsider subordinates according to intimacy, loyalty, and ability, and then treat them differently (Zheng, 2004; Ruan, 2018). Jiang and Zhang (2010) compared the differential leadership and categorization model of employees based on cultural specialty, and redefined differential leadership from the perspective of employees’ perception. The leader-member exchange (LMX) (Graen and Uhl-Bien, 1995) developed in the context of Western culture and differential leadership seem similar on the surface, but they have many differences. First, the cultural backgrounds of the two leadership styles are different. LPC-LMX is based on the social structure of equality between people in the West, the exchange relationship between leaders and employees is based on the law of fair exchange, while the differential leadership is based on the cultural context of Chinese humanism and relationship orientation, the leader is in the dominant position and the employee is in the subordinate position, the relationship between the two is not equal (Sikora and Ferris, 2014). Second, the classification criteria of the two leadership styles are different. LPC-LMX emphasizes employee ability, work interaction and value orientation; while differential leadership emphasizes the closeness and loyalty between employees and themselves. Third, the differential treatment of the two leadership styles is different. In the LPC-LMX, in-group employees with better leadership and exchange quality only show their trust and support at work; but differential leadership is not only limited to care at work, but also shows the family and emotions of their own employees, intimacy, care and trust in life, etc., and more communication and interaction with their own employees in private. Therefore, the leadership-member exchange theory under the background of Western culture and the differential leadership under the Chinese cultural background are completely different in nature. When discussing the differential treatment of leadership in Chinese enterprises and organizations, differential leadership is more culturally appropriate.

Deviant innovation behavior is a complex concept that consists of two very different factors: “deviance;” and “innovation.” Obedience to instructions is a basic requirement for participating in organizational work, but deviant behaviors ignore formal and informal rules and regulations and violate normative expectations in the workplace (Staw and Boettger, 1990; Tsui et al., 2000; Warren, 2003). Innovation is a creative process in which subordinates develop and practice innovative ideas, pursue value-added resources through updating technologies and methods, and finally produce innovative results that can play a role at a specific moment (Stein, 1953; Ford, 1996; Chen et al., 2017). Therefore, deviant innovation behavior takes “innovation” as the goal and “deviance” as the means. The rationality of the goal and the deviation of the behavior make it a special form of innovation behavior. Although scholars have different understandings regarding deviant innovation behavior, they concur that it aims to improve organizational interests and tries to assist innovation through deviant behavior, reflecting the non-traditional characteristics of organizational behavior (Galperin, 2002; Jiang, 2018). In conclusion, the present study defines deviant innovation behavior as follows: when organizational management and leadership authority become obstacles to innovation, if subordinates believe that their innovative ideas will bring benefits to the organization, they will choose to practice them through unconventional means, regardless of whether leaders approve or not, and perform innovative behavior that is inconsistent with organizational norms and leaders’ expectations.

Given that the leaders’ subordinate categorization model is dynamic, the relationship between leaders and subordinates resulting from differential leadership is not static (Thau et al., 2015). Outsider subordinates tend to establish good interaction and communication with leaders by improving relationships, showing their royalty, and enhancing their abilities. Outsider subordinates try to meet leaders’ expectations and gain their recognition through positive work performance, thus realizing the transformation from “outsider” to “insider,” in order to improve their social status and obtain more promotion opportunities. If employees realize that they can achieve the desired results by changing their behaviors, this will stimulate their innovative behaviors (Amabile et al., 2005). Accordingly, the first hypothesis is proposed:

**Hypothesis 1***:* Differential leadership has a positive effect on outsider subordinates’ deviant innovation behavior.

### Mediating effect of relative deprivation

Since Stouffer et al. (1949) first proposed the concept of “relative deprivation,” it has become an important research topic in psychology, sociology, politics, and economics, and an explicit definition and systematic theoretical framework have gradually emerged. Based on the different reference group selected, relative deprivation is classified into horizontal relative deprivation and longitudinal relative deprivation (Wang, 2000, 2007; Walker and Pettigrew, 2011; Xiong and Ye, 2016). The former is derived from individual comparison in the spatial dimension. It is a negative feeling of an individual induced by his/her weak situation, such as anger and dissatisfaction. The latter is derived from individual comparison in the temporal dimension, i.e., a comparison of the present situation with the past, future, or desired situation. It is a negative feeling induced by the incompatibility between individual value expectation and one’s ability. Accordingly, this study defines relative deprivation as (in the process of the horizontal or longitudinal comparison of individuals within the reference group) the subjective perception and emotional experience of anger and dissatisfaction induced by the differences between what is expected and what is actually received.

According to relative deprivation theory, when employees perceive unfair or discriminatory treatment in the workplace, this generates a sense of frustration and relative deprivation (Priesemuth and Taylor, 2016; Wan et al., 2016). Therefore, when leaders provide better material benefits, development opportunities, and social status to insider subordinates, it will send discriminatory signals to outsider subordinates that they are not valued and trusted by the organization, thus leading to outsider subordinates believing that they suffer more loss (Lin and Cheng, 2017; Wang et al., 2018). Underprivileged outsider subordinates often feel entitled to the same treatment and have a strong sense of deprivation because of their marginalized situation. Accordingly, the second hypothesis is proposed:

**Hypothesis 2:** Differential leadership has a positive effect on outsider subordinates’ relative deprivation.

The feeling of relative deprivation reflects people’s strong dissatisfaction with their situation and strong desires to change it. The generation and reinforcement of the feeling of relative deprivation provide the psychological drives and prerequisites for initiating action to compulsively correct “relative deprivation.” The purpose is often to break through class boundaries and achieve upward individual mobility through innovative ideas by those who are dissatisfied with the *status quo* (Mummendey et al., 1999). The feeling of relative deprivation is an underlying psychological experience that reflects the degree of social satisfaction of individuals or groups and the price that people have to pay to meet such needs, and it is a by-product of people’s efforts to change the *status quo* (Cheng and Chan, 2008). People who feel relative deprivation believe that they are entitled to fairer treatment, and that their ideas are feasible but that they lack the support that they should receive. Therefore, they may act without the leader’s approval, which results in deviant innovation behavior. Accordingly, the third hypothesis is proposed:

**Hypothesis 3:** Relative deprivation has a positive effect on outsider subordinates’ deviant innovation behavior.

Relative deprivation is the link between the external environment and individual behavior. Individuals assess the external environment through social comparison and experience anger, dissatisfaction, and other subjective perceptions due to the strong sense of unfairness (Guo and Zhang, 2014), resulting in changes in their attitude and behavior (Smith and Ortiz, 2002). Therefore, individuals will use a variety of conventional or unconventional means to work hard and pursue career development (Wang, 1988; Smith et al., 2012; Wan et al., 2016) in order to reduce the consequences of negative emotions (Bachleitner and Zins, 1999; Bennett and Robinson, 2000). Based on relative deprivation theory, Adams (1965) proposed equity theory regarding motivation for taking initiative in the workplace, which reflects people’s desire to improve the *status quo*. Moderate relative deprivation will lead to the expectation that individuals can realize their goals, having a positive impact on individuals’ internal psychological adaptation and external social adaptation (Gursoy and Kendall, 2006). Some scholars also believe that relative deprivation will improve individuals’ self-esteem (Han et al., 2017), and that employees may be motivated by expectations for a higher-status identity (Mummendey et al., 1999). Accordingly, the fourth hypothesis is proposed:

**Hypothesis 4:** Relative deprivation is the mediator between differential leadership and outsider subordinates’ deviant innovation behavior.

### Moderating effect of inner control personality

Rotter (1966) developed social learning theory based on Heider’s (1958) attribution theory, initially proposing internal and external loci of control. According to attribution theory, people with the internal control personality trait attribute the occurrence and outcome of events to internal subjective factors. Due to different attribution styles, people with internal and external control personality traits have significant differences in perception and behavior (Krenl, 1992), which can be introduced to explain the motivation and rules of people’s behavioral decision-making. Based on the literature, the present study defines the internal and external loci of control as a psychological perception that is used to assess whether an individual attributes the outcomes of an event to his/her own factors or external factors.

Previous studies have shown that attribution styles have a significant impact on the relationship between relative deprivation and behavior reactions (Smith and Ortiz, 2002). If individuals with a feeling of relative deprivation view it positively and face up to their difference with reference objects, they can take effective measures to narrow the gap. Regarding the relationship between the locus of control and relative deprivation, Crosby (1976) believed that different types of locus of control have different effects on individuals’ relative deprivation. Individuals with internal control personality traits tend to attribute the outcome of events to their own responsibility (Rotter, 1966), and they will seek opportunities for change to turn the disadvantageous situation into an advantageous one through self-criticism (Smith et al., 2012). Perceived control gives individuals confident self-awareness (Seligman and Marshak, 1990; Henderson and Zimbardo, 1999) and helps individuals face disadvantageous situations actively (Smith et al., 2012), thus leading to good response behaviors (Luthans, 2002) and realizing expectations for the future (Erez and Judge, 2001) and for inner satisfaction (Li, 2011). Therefore, although relative deprivation exists, individuals with internal control personality can turn it into an internal drive to change the disadvantageous situation by correctly analyzing it, leading to the desire for innovation, the pursuit of enhanced social status, and the expectation of making breakthroughs in their organization. Therefore, the fifth hypothesis is proposed:

**Hypothesis 5:** Internal control personality has a positive moderating effect on the relationship between relative deprivation and outsider subordinates’ deviant innovation behavior.

### Moderated mediator

The stress perception and response of individuals are obviously affected by their psychological control sources (Allen et al., 2008). Relative deprivation is the subjective perception and emotional experience of psychological stress owing to an individual putting herself/himself in a disadvantageous situation during social comparison. Psychological control sources also affect the response behavior of individuals in relation to the feeling of relative deprivation (Xiong and Ye, 2016). Individuals with internal control personality traits tend to assess the external environment from a positive perspective, focusing on the situation sources that cause stress and trying to solve problems through constructive strategies (Allen et al., 2003). Therefore, employees with internal control personality are less susceptible to external stress factors. They believe that, through active information seeking and improving work capacity, one can achieve an advantageous position in organizations quickly. Wu and He (2015) verified the positive influence of the internal control personality trait on organizational citizenship behaviors. Deviant innovation is an extra-role activity with high risk, which requires employees to actively capture opportunities, set goals and strategies, and take actions (Zhang, 2016). The internal control personality trait can help employees improve their motivation to obtain rewards by changing their behavior (Gao et al., 2014). It promotes ambitions by satisfying people’s needs for self-actualization, thereby improving work efficiency. Therefore, individuals will adopt the above behaviors and perception modes to eliminate psychological imbalances, strive to improve self-conditions, and narrow the gap with reference objects. Accordingly, the sixth hypothesis is proposed:

**Hypothesis 6:** Internal control personality plays a moderated mediating role in the process of differential leadership indirectly influencing outside subordinates’ deviant innovation behavior through the mediating effect of relative deprivation.

**FIGURE 1:**
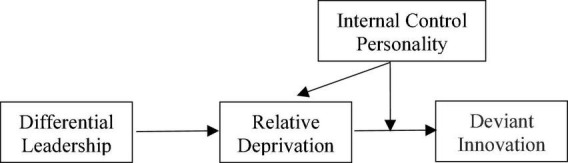
Theoretical model.

This study’s theoretical model is depicted in Figure 1.

## Research design

### Data

The present study used a questionnaire, and sample data were collected from organizations in Hainan province, China. The questionnaire addressed employees’ assessments regarding differential leadership, feelings of relative deprivation, internal control personality, and deviant innovation behavior. A total of 300 questionnaires were distributed, of which 243 were valid (an effective response rate of 81.00%). Regarding the valid responses, the following key demographic distributions were found: 51.03% of employees were male and 48.97% were female; 65.43% of employees were aged 31–40 years; 11.93% of employees had a junior college degree and 63.79% had a Bachelor’s degree; 68.31% of employees had been working in their current organization for 3–5 years; 19.75% of employees were from state-owned enterprises, 14.81% from private enterprises, 21.40% from foreign ventures, 22.63% from joint ventures, and 21.40% from other types of enterprises; and 73.66% of respondents were business employees.

### Variables

This study adopted the Vineland Social Maturity Scale, and all items in the scale were scored on a seven-point Likert scale to avoid many neutral answers from respondents. The description of the variables is as follows:

• *Differential leadership*. We adopted a 14-item scale developed by Jiang and Zhang (2010) (example item: “I spend more time on individual guidance”). The reliability of the scale is 0.877.
• *Relative deprivation*. We adopted a five-item scale developed by Tropp and Wright (1999) (example item: “I perceive I have been treated unfairly”). The reliability of the scale is 0.863.
• *Internal control personality*. We adopted an eight-item scale developed by Spector (1988) (example item: “Getting the job you want mostly relies on luck”). The reliability of the scale is 0.912.
• *Deviant innovation.* We adopted a five-item scale developed by Criscuolo et al. (2014) (example item: “I like to think of new ideas beyond my duty”). The reliability of the scale is 0.870.
• *Control variables.* We selected several demographic variables, including gender, age, education, work seniority, organization type, and occupation type, that may affect differential leadership, relative deprivation, internal control personality, and deviant innovation.

## Analysis and results

### Common method bias test and confirmatory factor analysis

Harman’s single factor analysis was adopted in this study to conduct exploratory factor analysis on all items of the four variables. The results revealed a KMO value of 0.860, a Bartlett’s Chi-square test of sphericity value of 5,437.908, and a *p*-value less than 0.001. In addition, the extracted four common factors were consistent with the number of variables set in this study, and the degree of variance of the first variance was 29.498%, which is lower than the critical value of 50%. Therefore, there is no serious common method bias in the data of this study.

In this study, confirmatory factor analysis was adopted to test the discriminative validity of differential leadership, relative deprivation, internal control personality, and deviant innovation behavior. As shown in Table 1, compared with the other three models, the four-factor model has the best fitting effect, and each indicator reaches or approaches the indicator requirements, among which χ^2^/df = 1.979, CFI = 0.915, TLI = 0.907, RMSEA = 0.064, and IFI = 0.916. In summary, the four variables in this study have good discriminative validity.

**TABLE 1:** AMOS confirmatory factor analysis.

Models	χ^2^	df	χ^2^/df	RMSEA	CFI	IFI	TLI
Four-factor model D	890.725	450	1.979	0.064	0.915	0.916	0.907
Three-factor model C	1,471.747	457	3.220	0.096	0.805	0.807	0.789
Two-factor model B	1,808.074	460	3.931	0.110	0.741	0.743	0.721
One-factor model A	1,977.352	461	4.289	0.117	0.709	0.711	0.687

Model A: differential leadership + relative deprivation + internal control personality + deviant innovation. Model B: differential leadership + relative deprivation + internal control personality; deviant innovation. Model C: differential leadership; relative deprivation + internal control personality; deviant innovation. Model D: differential leadership; relative deprivation; internal control personality; deviant innovation.

### Descriptive statistical analysis

The results of the correlation analysis of the research are shown in Table 2, in which the mean value, standard deviation, and correlation coefficient of the variables are given. Differential leadership has a significant positive correlation with deviant innovation (*r* = 0.532, *p* < 0.01), and with relative deprivation (*r* = 0.319, *p* < 0.01). There is a significant positive correlation between relative deprivation and deviant innovation (*r* = 0.282, *p* < 0.01).

**TABLE 2:** Descriptive statistical results for the variables.

Variable	Mean	SD	1	2	3	4	5	6	7	8	9	10
1. Gender	1.49	0.501	1									
2. Age	2.93	0.901	0.044	1								
3. Education	2.96	0.926	–0.108	−0.197**	1							
4. Work seniority	2.91	0.891	–0.076	–0.029	0.096	1						
5. Organization types	3.11	1.420	0.028	–0.039	–0.012	0.028	1					
6. Occupation types	3.06	0.780	–0.104	0.088	–0.014	0.013	–0.036	1				
7. Differential leadership	4.10	0.932	0.014	0.036	–0.047	–0.075	0.035	–0.090	1			
8. Relative deprivation	4.32	1.237	–0.033	0.023	0.019	0.030	–0.016	0.019	0.319**	1		
9. Internal control personality	4.09	0.923	–0.070	0.024	0.034	0.050	–0.072	0.077	0.402**	0.179**	1	
10. Deviant innovation	4.02	1.010	–0.053	0.013	0.011	–0.019	–0.033	–0.007	0.532**	0.282**	0.611**	1

Symbol ** denotes *p* < 0.01.

### Hypothesis testing

Baron and Kenny (1986)’s hierarchical regression method was adopted in this study to analyze the utility of the mediating and moderating variables. The steps followed to explore the mediating role of relative deprivation are as follows. First, six demographic control variables (gender, age, education, work seniority, organization type, and occupation type) were put into the regression equation. Second, an independent variable (differential leadership) was introduced into the equation. Finally, we tested the mediating effect of relative deprivation on the relationship between differential leadership and deviant innovation behavior.

The steps followed to explore the moderating effect of internal control personality are as follows. First, we put six control variables into the regression equation. Second, relative deprivation and internal control personality were introduced into the regression equation. Third, the interaction terms of relative deprivation and internal control personality were put into the regression equation to explore their influence.

#### Main effect and mediating effect tests

As shown in Table 3, there is a significant positive correlation between differential leadership and deviant innovation (β = 0.585, *p* < 0.01), thus verifying *H1*. There is a significant positive correlation between differential leadership and relative deprivation (β = 0.437, *p* < 0.01), which verifies *H2*. When the mediating variable of relative deprivation is introduced in the relationship between differential leadership and deviant innovation, there is a significant positive correlation between relative deprivation and deviant innovation (β = 0.230, *p* < 0.01), thus verifying *H3*. In addition, the positive effect of differential leadership on deviant innovation is weakened by introducing the mediating variable of relative deprivation (β changed from 0.585 to 0.543, *p* < 0.01), indicating that relative deprivation plays a mediating role in the relationship between differential leadership and deviant innovation, thus verifying *H4*.

**TABLE 3:** Mediating effect of relative deprivation (*n* = 243).

Variable	Relative deprivation		Deviant innovation behavior		
	Model 1	Model 2	Model 3	Model 4	Model 5
Gender	–0.071	–0.065	–0.104	–0.096	–0.098
Age	0.037	0.020	–0.003	0.011	–0.005
Education	0.025	0.040	0.031	0.005	0.027
Work seniority	0.037	0.070	0.018	–0.034	0.011
Organization types	–0.012	–0.022	–0.035	–0.019	–0.033
Occupation types	0.021	0.069	0.045	–0.024	0.039
Differential leadership		0.437**	0.585**		0.543**
Relative deprivation				0.230**	0.097*
*F*	0.127	4.126***	13.798***	3.069***	12.770***
*R* ^2^	0.003	0.109	0.291	0.084	0.304
Δ*R*^2^		0.106	0.286	0.079	0.013

Symbol * denotes *p* < 0.05, ** denotes *p* < 0.01, *** denotes *p* < 0.001.

In order to further test the mediating effect of relative deprivation, this study adopted the PROCESS macro program to conduct bootstrap analysis. The results showed that the mediating effect of relative deprivation with a bootstrap 95% confidence interval is (0.001, 0.089), excluding 0, which indicates that the mediating effect of relative deprivation is significant, and the effect value is 0.042 (*SE* = 0.022).

#### Moderating effect of internal control personality

**FIGURE 2:**
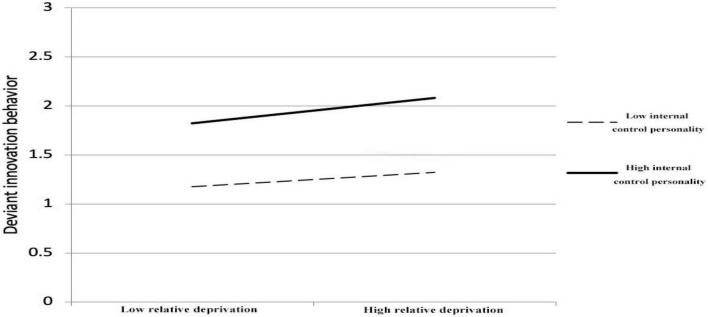
Moderating effect of internal control personality.

As shown in Table 4, the regression coefficient of the interaction term of relative deprivation and internal control personality is significant (β = 0.113, *p* < 0.05), indicating that internal control personality has a moderating effect on the relationship between relative deprivation and deviant innovation, thus verifying *H5*. Figure 2 illustrates the moderating effect of internal control personality on the relationship between relative deprivation and deviant innovation. As shown in Figure 2, relative deprivation has a more significant impact on the deviant innovation behavior of employees with high internal control personality compared to those with low internal control personality.

**TABLE 4:** Moderating effect of internal control personality (*n* = 243).

Variable	Deviant innovation behavior		
	Model 6	Model 7	Model 8
Gender	–0.112	–0.036	–0.040
Age	0.019	–0.003	–0.001
Education	0.010	–0.012	–0.012
Work seniority	–0.026	–0.061	–0.054
Organization types	–0.022	0.008	0.001
Occupation types	–0.019	–0.073	–0.068
Relative deprivation		0.147**	0.150**
Internal control personality		0.641**	0.646**
Relative deprivation × internal control personality			0.113*
*F*	0.192	20.364***	19.263***
*R* ^2^	0.005	0.410	0.427
Δ*R*^2^	0.005	0.406	0.016

Symbol * denotes *p* < 0.05, ** denotes *p* < 0.01, *** denotes *p* < 0.001.

#### Test of moderated mediating effects

This study adopted the PROCESS macro program developed by Hayes and Preacher (2013) to test the moderated mediating effect, with a 95% confidence interval, adopting Mean ± SD to distinguish the mediating effects of relative deprivation under different levels of internal control personality: high; medium; and low. As shown in Table 5, the mediating effect of relative deprivation under high internal control personality is significant and strong, and the effect value is 0.074 (*SE* = 0.028). The mediating effect of relative deprivation under low internal control personality is comparatively weaker and insignificant, and the effect value is 0.002 (*SE* = 0.024). Therefore, the partial mediating effect of relative deprivation on the relationship between differential leadership and deviant innovation is affected by internal control personality, and there is a moderated mediating effect. The higher the level of internal control personality, the stronger the mediating effect of relative deprivation, which verifies *H6*.

**TABLE 5:** Test of moderated mediating effects.

Effect	Mediating path	Internal control personality	Effect value	*SE*	Boot LLCI	Boot ULCI
Indirect effect	Relative deprivation	High	0.074	0.028	0.028	0.143
		Middle	0.038	0.019	0.005	0.081
		Low	0.002	0.024	−0.048	0.049

## Conclusion and research prospects

### Conclusion

Based on relative deprivation theory, this study has explored the mechanism of differential leadership influencing employees’ deviant innovation behavior, as well as the mediating effect of relative deprivation and the moderating effect of internal control personality. The results verify all six hypotheses, confirming that: differential leadership has a positive effect on outsider subordinates’ deviant innovation behavior (*H1*); differential leadership has a positive effect on outsider subordinates’ relative deprivation (*H2*); relative deprivation has a positive effect on outsider subordinates’ deviant innovation behavior (*H3*); relative deprivation is the mediator between differential leadership and outsider subordinates’ deviant innovation behavior (*H4*); internal control personality has a positive moderating effect on the relationship between relative deprivation and outsider subordinates’ deviant innovation behavior (*H5*); and internal control personality plays a moderated mediating role in the process of differential leadership indirectly influencing outside subordinates’ deviant innovation behavior through the mediating effect of relative deprivation (*H6*).

### Theoretical significance

First, this study has explored the influence of differential leadership on employees’ deviant innovation behaviors, extending research on the antecedents of outsider subordinates’ deviant innovation behaviors. Most previous studies have focused on the consequences of employees’ deviant innovation behaviors, while the exploration of its logical deconstruction is relatively scarce. Therefore, a systematic study on the influence of leadership style on employees’ deviant innovation behaviors is lacking. Starting with the construction of “China’s own leadership values,” this study has conducted empirical testing on the mechanism of differential leadership influencing employees’ deviant innovation behavior, analyzing the internal relationship between them.

Second, most previous studies on differential leadership have focused on its negative influences, while the positive effects have largely been ignored. This study has explored differential leadership and its effectiveness in the context of Chinese culture, which enriches the theoretical study of differential leadership. By incorporating differential leadership into research on employees’ deviant innovation behavior, this study strengthens the theoretical framework of employees’ deviant innovation behavior in the context of Chinese culture, providing new ideas for related research and expanding the theoretical research perspective.

Finally, based on relative deprivation theory, this study has introduced the relative deprivation of outsider subordinates as a mediating variable to explore the influence of differential leadership on employees’ deviant innovation, which enriches research on the mediating mechanism of relative deprivation and provides a theoretical reference for further exploration of the causes of deviant innovation. In addition, based on attribution theory, this study has used internal control personality as a moderating variable to systematically explain the moderating mechanism of the influence of differential leadership on employees’ deviant innovation behavior. It thus expands the boundary conditions for the generation of deviant innovation, enriches the related research pertaining to attribution theory, and provides a theoretical reference point for optimizing deviant innovation behavior.

#### Practical significance

First, in view of the important influence of differential patterns in the economy, studying differential leadership and its effectiveness has important practical value in optimizing differential leadership. The findings help understand the influence and effectiveness of differential leadership in the context of management localization in China, and provide a reference point for the appropriate adjustment of leadership style and the cultivation of managers with differential leadership skills, in order to guide management styles and thinking modes to fit sustainable development.

Second, this study has explored the influence mechanism of differential leadership on employees’ deviant innovation, providing practical reference for the effective optimization of employees’ deviant innovation behavior. The finding helps leaders to fully understand the path of differential leadership in improving employees’ deviant innovation behavior and provides insights into different strategies to improve employees’ innovation. The paper provides a theoretical foundation for leaders to motivate employees to break their shackles and effectively govern deviant innovative behaviors, providing empirical support for improving enterprises’ innovation values and promoting sustainable development, thus realizing the improvement of the overall innovation performance of organizations.

Finally, this study has explored the mediating effect of relative deprivation between differential leadership and deviant innovation. This helps managers to correctly understand employees with feelings of relative deprivation and to provide the necessary psychological counseling and encouragement in order to address feelings of relative deprivation. Although relative deprivation is an unpleasant feeling, the findings indicate that moderate relative deprivation can motivate employees to work harder to change their situation. Therefore, managers need to pay attention to the psychological status of employees and give full play to the positive role of relative deprivation.

### Limitations and research prospects

There are some limitations in this study that should be addressed in future studies. First, the scales adopted in the questionnaire were translated from foreign scales. However, due to cultural differences between China and foreign countries, there may be some limitations in applicability. Future studies should further develop localization scales and improve the applicability in China. Second, all data in this study came from the self-assessment of employees; thus, common method bias may exist. Future studies could adopt the pairing method to collect data to test the hypotheses. Third, this study only explored the influence mechanism of differential leadership on employees’ deviant innovation behavior from the perspective of outsider subordinates. In the future, studies could compare the different influences of differential leadership on employee behavior both from the perspectives of insiders and outsiders. Finally, this study only took China as the research object. It does not consider the differential impact of organizational culture. Subsequent research could consider the cross-cultural applicability of differential leadership theory and promote the localization theory of China to other regions.

## Data availability statement

The original contributions presented in this study are included in the article/supplementary material, further inquiries can be directed to the corresponding author.

## Author contributions

YZ, MW, and QH contributed to the conception of the study. MW and LZ performed the experiment. LZ contributed significantly to analysis and manuscript preparation. JL, YZ, and LZ performed the data analyses and wrote the manuscript. JL and YZ helped to perform the analysis with constructive discussions. All authors contributed to the article and approved the submitted version.
